# Impaired heart rate recovery is associated with new-onset atrial fibrillation: a prospective cohort study

**DOI:** 10.1186/1471-2261-9-11

**Published:** 2009-03-12

**Authors:** Thomas M Maddox, Colleen Ross, P Michael Ho, David Magid, John S Rumsfeld

**Affiliations:** 1 Cardiology Section, Denver VAMC, Denver, CO, USA; 2 Department of Medicine (Cardiology), University of Colorado Denver, Denver, CO, USA; 3 Institute for Health Research, Kaiser Permanente Colorado, Denver, CO, USA

## Abstract

**Background:**

Autonomic dysfunction appears to play a significant role in the development of atrial fibrillation (AF), and impaired heart rate recovery (HRR) during exercise treadmill testing (ETT) is a known marker for autonomic dysfunction. However, whether impaired HRR is associated with incident AF is unknown. We studied the association of impaired HRR with the development of incident AF, after controlling for demographic and clinical confounders.

**Methods:**

We studied 8236 patients referred for ETT between 2001 and 2004, and without a prior history of AF. Patients were categorized by normal or impaired HRR on ETT. The primary outcome was the development of AF. Cox proportional hazards modeling was used to control for demographic and clinical characteristics. Secondary analyses exploring a continuous relationship between impaired HRR and AF, and exploring interactions between cardiac medication use, HRR, and AF were also conducted.

**Results:**

After adjustment, patients with impaired HRR were more likely to develop AF than patients with normal HRR (HR 1.43, 95% confidence interval (CI) 1.06, 1.93). In addition, there was a linear trend between impaired HRR and AF (HR 1.05 for each decreasing BPM in HRR, 95% CI 0.99, 1.11). No interactions between cardiac medications, HRR, and AF were noted.

**Conclusion:**

Patients with impaired HRR on ETT were more likely to develop new-onset AF, as compared to patients with normal HRR. These findings support the hypothesis that autonomic dysfunction mediates the development of AF, and suggest that interventions known to improve HRR, such as exercise training, may delay or prevent AF.

## Background

Atrial fibrillation (AF) is a common condition in the U.S., with over 75,000 new cases annually.[1] Though its etiology is multi-factorial, prior mechanistic studies have demonstrated that autonomic dysfunction in general, and decreased parasympathetic function in particular, may play a significant role in its development. [2-5] Thus, clinical tests that measure autonomic dysfunction may identify patients at a higher likelihood of developing AF, providing further support to the hypothesis that autonomic dysfunction is a mediator of the condition.

Exercise treadmill testing (ETT) is a widely available clinical tool that provides an assessment of autonomic dysfunction via measurement of heart rate recovery (HRR). Impaired HRR, defined as a decrease of less than 12 beats one minute after peak exercise, is associated with decreased parasympathetic activity, and thus may identify patients more likely to develop AF.

Accordingly, we hypothesized that patients with impaired HRR on ETT were more likely to develop new-onset AF as compared to those patients with normal HRR, after adjustment for demographic and clinical factors associated with AF. Demonstration of an association between AF and impaired HRR during ETT would add to the basic physiologic, animal, and clinical observation studies supporting the concept of the autonomic nervous system as a potential mediator of AF.[2]

## Methods

### Study population and data collection

We examined a consecutive, prospective cohort of patients without a prior diagnosis of AF or atrial flutter who were referred for ETT between July 2001 and June 2004. All patients were enrolled in Kaiser Permanente of Colorado (KPCO), an integrated, nonprofit managed care organization (MCO) that provides medical services to more than 475,000 members in the Denver/Boulder/Colorado Springs, Colorado metropolitan area. Inclusion criteria for the study included a minimum of 12 months of KPCO enrollment prior to the index ETT, no prior diagnosis of AF or atrial flutter in either claims or medical record data prior to or during the index ETT, and no use of class I or III anti-arrhythmic drugs at the time of the index ETT.

Prior to exercise testing, a structured history and medical record review were performed to document symptoms, past medical history, medication use, cardiac risk factors, and prior cardiac events and procedures. Additional co-morbidity data (e.g., cerebrovascular and peripheral vascular disease) were obtained from the KPCO databases. Symptom-limited exercise treadmill testing was performed according to standardized protocols, with the Bruce protocol used in 85% of tests. After achievement of peak exercise, all patients underwent a 1 minute 'cool-down' period by walking on the treadmill at 1.0 mile per hour, as specified in prior research on HRR.[6] During each exercise stage and recovery stage, symptoms (e.g., chest pain, shortness of breath, fatigue, dyspnea, and dizziness), blood pressure, heart rate, cardiac rhythm, and metabolic equivalents (METs) were recorded. The reasons for termination of exercise, including dyspnea, fatigue, chest pain, ischemic ST changes, marked elevation in blood pressure, or ventricular ectopy were recorded. Achieving target heart rate alone was not used as a justification for terminating exercise. All clinical and exercise data were entered contemporaneously into an electronic database. For patients undergoing multiple treadmill tests during this period, only the first treadmill test was considered in the analyses.

### Variables

Our independent predictor variable of interest was abnormal HRR, defined as a decrease of 12 beats/min or less from peak exercise heart rate and one minute into recovery.[6] Our primary outcome of interest was the occurrence of new-onset AF during the follow-up period, as determined by the presence of a 427.31 ICD-9 code in a facility claim or clinic visit. Follow-up information was available after the exercise test on 99% of patients through October 31, 2005. Patients who died or disenrolled from Kaiser during the observation period were censored at the time of disenrollment in the analyses.

### Statistical Analysis

After categorization of patients by normal or abnormal HRR, baseline characteristics were compared using the chi-square test for categorical variables and Wilcoxon rank-sum scores for continuous variables. Next, unadjusted comparisons of time to new-onset AF by normal or impaired HRR were performed using the log-rank test and graphically represented by Kaplan-Meier curves. Then, multivariable Cox proportional hazard models were constructed to determine the association between impaired HRR and new-onset AF, adjusting for covariates. Co-variates entered into the multivariable model were selected based on *a priori *research or those identified in bi-variate analyses as associated with AF. Selected co-variates included age, gender, hypertension, congestive heart failure, coronary artery disease, mitral valve disease, obesity, and diabetes. [7-12] Age and gender, then clinical variables, were entered into the model sequentially to assess for incremental effects on the primary association and potential co-linearity. Cox proportional hazards assumptions were tested using Schoenfeld residuals.

In order to determine if the severity of impaired HRR was associated with new-onset AF in a dose-response relationship, we conducted a secondary analysis examining abnormal HRR values (HRR ≤ 12) as a continuous variable and its linear association with new-onset AF In addition, since medication use (ACEI, beta-blocker, and statin medications) may affect the development of new-onset AF, [13-16] we also conducted a secondary analysis of the association between HRR and new-onset AF incorporating their use.

The study was approved by the Kaiser Permanente Colorado Institutional Review Board. All analyses were performed using the SAS statistical package version 9.1 (SAS Institute, Cary, NC).

## Results

### Descriptives

**Figure 1:**
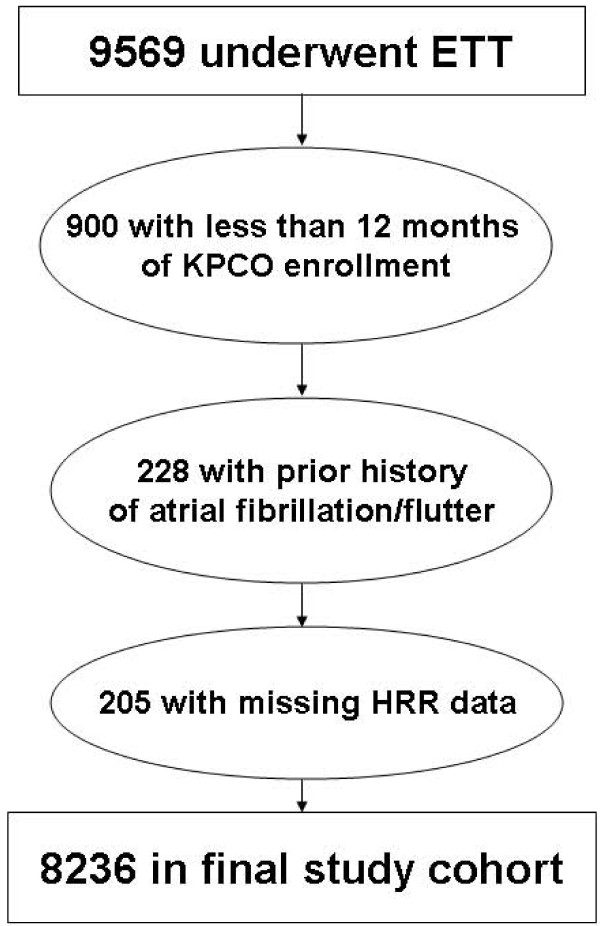
**Study cohort creation**.

Of 9569 patients undergoing ETT during the study period, 1128 patients did not meet the inclusion criteria or had missing HRR data, resulting in 8236 patients for the study cohort (Figure 1). Patient demographics, clinical history, and ETT indications and characteristics are listed in Table 1. In general, patients with impaired HRR were older, female, and had a higher prevalence of cardiac risk factors, pre-existing CAD, and other co-morbidities as compared to patients with normal HRR. The median length of follow-up was 3.1 years.

**Table 1:** Baseline characteristics of patients by heart rate recovery (HRR) on exercise treadmill testing (n = 8236)

	**Impaired HRR n = 2425**	**Normal HRR n = 5811**	**P value**
**Demographics**			
Age, median (25th, 75th percentiles), y	63 (54, 70)	54 (47, 63)	< 0.0001
Male, No. (%)	1199 (49.4)	3113 (53.6)	< 0.001
			
**ETT indications**			< 0.0001
Chest pain	451 (18.6)	984 (16.9)	
Atypical chest pain	920 (37.9)	2953 (50.8)	
Risk stratification	200 (8.3)	354 (6.1)	
Screening	272 (11.2)	580 (10.0)	
Other	582 (24.0)	940 (16.2)	
			
**Clinical History**			
Hypertension, No. (%)	1552 (64)	2487 (42.8)	< 0.0001
Diabetes Mellitus, No. (%)	546 (22.5)	619 (10.7)	< 0.0001
Lipid disease, No. (%)	1677 (69.2)	3611 (62.1)	< 0.0001
Current or recent smoking, No. (%)	393 (16.2)	741 (12.8)	< 0.0001
Obese (BMI > = 30), No. (%)	1026 (42.3)	1736 (29.9)	< 0.0001
Coronary artery disease, No. (%)	450 (18.6)	629 (10.8)	< 0.0001
Congestive heart failure, No. (%)	74 (3.1)	43 (0.7)	< 0.0001
COPD, No. (%)	237 (9.8)	145 (2.5)	< 0.0001
Mitral valve disease, No. (%)	58 (2.4)	99 (1.7)	0.04
			
**Medications**			
ACE inhibitor use, No. (%)	596 (24.6)	800 (13.8)	< 0.0001
Beta-blocker use, No. (%)	743 (30.6)	1213 (20.9)	< 0.0001
- within 72 hours of ETT	558 (23)	929 (16)	< 0.0001
Statin use, No. (%)	714 (29.4)	1213 (20.9)	< 0.0001
Calcium channel blocker use, No. (%)	195 (8)	214 (3.7)	< 0.0001

ACE – angiotensin converting enzyme

BMI – body mass index

COPD – chronic pulmonary obstructive disease

### Unadjusted and adjusted associations

**Figure 2:**
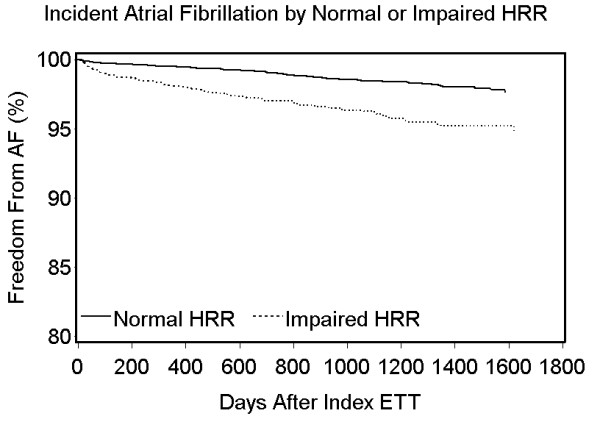
**Unadjusted Kaplan-Meier curve of new-onset atrial fibrillation by heart rate recovery on exercise treadmill testing (n = 8236)**.

During the study period, 99 (4.1%) patients with impaired HRR and 93 (1.6%) patients with normal HRR had new-onset AF (Figure 2, log-rank p-value < 0.0001). After adjustment for age and gender, impaired HRR was significantly associated with a higher risk of new-onset AF (hazard ratio (HR) 1.55, 95% confidence interval (CI) 1.15, 2.08), as compared to patients with normal HRR (Table 2). After adjustment for age, gender, and clinical factors, impaired HRR remained significantly associated with a higher risk of new-onset AF (HR 1.43, 95% CI 1.06, 1.93), as compared to patients with normal HRR (Table 2).

**Table 2:** Adjusted Hazard Ratios for New-Onset AF

**Independent variable**	**Hazard Ratio (95% CI) for New-Onset AF, controlling for age and gender**	**Hazard Ratio (95% CI) for New-Onset AF, controlling for age, gender, and clinical history**
**Impaired HRR**	**1.55 (1.15, 2.08)**	**1.43 (1.06, 1.93)**
Age (years)	1.09 (1.07, 1.1)	1.09 (1.07, 1.1)
Male gender	2.54 (1.85, 3.47)	2.65 (1.93, 3.65)
Hypertension		1.43 (1.03, 1.98)
Congestive heart failure		2.9 (1.56, 5.38)
Coronary artery disease		0.59 (0.4, 0.88)
Mitral valve disease		1.28 (0.62, 2.64)
Obesity		1.02 (0.74, 1.4)
Diabetes		1.15 (0.8, 1.65)

### Secondary analyses

In secondary analyses, there was a trend towards a linear relationship between impaired HRR and new-onset AF (HR 1.05 for each decreasing BPM in HRR, 95% CI 0.99, 1.11), p-value = 0.08). In addition, the addition of ACEI, beta-blocker, or statin use did not significantly change the primary association between impaired HRR and new-onset AF among patients (data not shown).

## Discussion

In this large, community-based cohort study, we found that patients with an impaired HRR on ETT were more likely to experience new-onset AF than patients without impaired HRR, independent of demographic and clinical factors. We also demonstrated a trend between increasing severity of HRR and new-onset AF, and that adjustment for ACEI, beta-blocker, or statin use did not alter the primary association. This information provides further clinical support to prior mechanistic studies demonstrating associations between autonomic dysfunction and AF, and identifies ETT as an additional tool to explore this relationship. Moreover, the results of this study suggest that patients found to have impaired HRR on ETT are at risk for the development of AF.

By measuring HRR, ETT appears to provide insight into the autonomic response to exercise, abnormalities of which have also been demonstrated to independently predict adverse cardiac outcomes.[17,18] HRR, sometimes termed vagal reactivation, is related to the capacity of the cardiovascular system to reverse those autonomic nervous system adaptations that occur during exercise, and abnormalities in HRR are believed to reflect imbalances in autonomic tone, and vagal tone in particular.[19] Imbalances in autonomic tone, and parasympathetic abnormalities in particular, have been demonstrated to facilitate the development of AF. [2-5] Accordingly, our demonstration of an association between impaired HRR and AF adds to the basic physiologic, animal, and clinical observation studies in supporting the concept of the autonomic nervous system as a potential mediator of AF.[2]

Prior studies have demonstrated impaired HRR is a valuable prognostic tool for adverse events, such as all-cause mortality and cardiovascular events.[6,20-26] However, to our knowledge, it has not previously been shown to be associated with AF. By illustrating this association, our study further underscores the value of ETT as a clinical tool. Demonstrating associations between impaired HRR and new-onset AF may also provide potential targets for AF prevention. For example, prior studies have demonstrated that, among patients with coronary artery disease, exercise training enhances vagal tone, improves HRR after exercise, and reduces morbidity.[27] In addition, a recent study demonstrated that light to moderate physical activity was associated with a lower incidence of AF.[28] The findings of our study provide a potential link between these prior observations and suggest several future avenues for research to understand and manage this increased risk for incident AF, including additional tests for autonomic dysfunction to evaluate these patients (e.g. heart rate variability), and the evaluation of interventions to prevent or delay the development of AF (e.g. intensive medical therapies, exercise training).[28,29] In addition, the prognostic information provided by HRR, which is routinely collected by most non-invasive laboratories, also argues for its consistent inclusion in ETT interpretation reports.

### Study limitations

This study has several potential limitations. First, our population was drawn from a single MCO health care system in Colorado referred specifically for ETT. Although this may limit the generalizability of our findings, the MCO is a large integrated healthcare delivery system, with broad representation of the state's population. In addition, patients referred for ETT are more likely to be older and have more cardiac-related co-morbidities than the general population, making their likelihood of AF development higher and their subsequent need for its detection and treatment greater. Second, we were unable to determine whether or not new-onset AF represented paroxysmal, persistent, or permanent AF. Future studies should seek to determine if HRR is associated with a particular form of AF. Finally, as with all observational studies, we are unable to definitively conclude whether this association truly represents causation.

## Conclusion

In conclusion, we found that impaired HRR was significantly associated with a higher likelihood of new-onset AF, independent of patient and clinical factors. Future research to validate these findings in other populations, explore the mechanistic links between impaired HRR and AF, and understand whether patients with impaired HRR benefit from interventions, such as exercise training, to delay or prevent new-onset AF are needed.

## Competing interests

The authors declare that they have no competing interests.

## Authors' contributions

TMM conceived and designed the study, conducted the analysis, and drafted the manuscript. CR conducted the statistical analysis. PMH, DM, and JSR participated in the design of the study and provided important intellectual contributions in the drafting of the manuscript. All authors have read and approved the final manuscript.

## Pre-publication history

The pre-publication history for this paper can be accessed here:


